# The clinical and mutational spectrum of B3GAT3 linkeropathy: two case reports and literature review

**DOI:** 10.1186/s13023-019-1110-9

**Published:** 2019-06-13

**Authors:** Marlies Colman, Tim Van Damme, Elisabeth Steichen-Gersdorf, Franco Laccone, Sheela Nampoothiri, Delfien Syx, Brecht Guillemyn, Sofie Symoens, Fransiska Malfait

**Affiliations:** 1 Center for Medical Genetics, Ghent University and Ghent University Hospital, 0K5, Corneel Heymanslaan 10, B-9000 Ghent, Belgium; 2 Department of Pediatrics, Medical University of Innsbruck, Innsbruck, Austria; 3 Institute of Medical Genetics, Vienna, Austria; 4 Department of Pediatric Genetics, Amrita Institute of Medical Sciences & Research Centre, Kerala, India

**Keywords:** Connective tissue, Glycosaminoglycans, GAG Linkeropathies, B3GAT3, Genotype, Phenotype

## Abstract

**Background:**

Proteoglycans are large and structurally complex macromolecules which can be found in abundancy in the extracellular matrix and on the surface of all animal cells. Mutations in the genes encoding the enzymes responsible for the formation of the tetrasaccharide linker region between the proteoglycan core protein and the glycosaminoglycan side chains lead to a spectrum of severe and overlapping autosomal recessive connective tissue disorders, collectively coined the ‘glycosaminoglycan linkeropathies’.

**Results:**

We report the clinical findings of two novel patients with a complex linkeropathy due to biallelic mutations in *B3GAT3*, the gene that encodes glucuronosyltransferase I, which catalyzes the addition of the ultimate saccharide to the linker region. We identified a previously reported c.667G > A missense mutation and an unreported homozygous c.416C > T missense mutation. We also performed a genotype and phenotype-oriented literature overview of all hitherto reported patients harbouring *B3GAT3* mutations. A total of 23 patients from 10 families harbouring bi-allelic mutations and one patient with a heterozygeous splice-site mutation in *B3GAT3* have been reported. They all display a complex phenotype characterized by consistent presence of skeletal dysplasia (including short stature, kyphosis, scoliosis and deformity of the long bones), facial dysmorphology, and spatulate distal phalanges. More variably present are cardiac defects, joint hypermobility, joint dislocations/contractures and fractures. Seven different *B3GAT3* mutations have been reported, and although the number of patients is still limited, some phenotype-genotype correlations start to emerge. The more severe phenotypes seem to have mutations located in the substrate acceptor subdomain of the catalytic domain of the glucuronosyltransferase I protein while more mildly affected phenotypes seem to have mutations in the NTP-sugar donor substrate binding subdomain.

**Conclusions:**

Loss-of-function mutations in *B3GAT3* are associated with a complex connective tissue phenotype characterized by disproportionate short stature, skeletal dysplasia, facial dysmorphism, spatulate distal phalanges and -to a lesser extent- joint contractures, joint hypermobility with dislocations, cardiac defects and bone fragility. Based on the limited number of reported patients, some genotype-phenotype correlations start to emerge.

## Background

Proteoglycans (PG) are large and structurally complex macromolecules which can be found in abundancy in the extracellular matrix and on the surface of all animal cells. PG are involved in a wide variety of functions such as cell-cell communication, cell-matrix interactions and cell growth and differentiation. PGs consist of a core protein with one or multiple glycosaminoglycan (GAG) sidechains covalently attached to it. GAGs are composed of repeating disaccharides consisting of an amino sugar (N-acetylglucosamine [GlcNAc] or N-acetylgalactosamine [GalNAc]) and an uronic acid (glucuronic [GlcA] or iduronic acid [IdoA]). The PG superfamily is subdivided into two major groups: heparan sulfate (HS) and chondroitin sulfate (CS)/dermatan sulfate (DS) PGs [1–3]. Before the polymerization of these HS and CS/DS chains, the synthesis of a linker region consisting of four saccharides is obligatory. This is a stepwise process requiring the coordinated action of specific transferases. First, a xylose unit is transferred onto a serine residue of the core protein by xylosyltransferase s I/II (encoded by *XYLT1* and *XYLT2*), followed by the addition of two galactose units by galactosyltransferase I (β4GalT7, encoded by *B4GALT7*) and galactosyltransferase II (β3GalT6, encoded by *B3GALT6*), respectively. The formation of the linker region is completed by the transfer of glucuronic acid catalysed by glucuronosyltransferase I (GlcAT-I, encoded by *B3GAT3*) [4–9].

Biallelic mutations in all genes encoding these linker region enzymes have been identified, leading to a spectrum of overlapping autosomal recessive multisystemic disorders, the ‘GAG linkeropathies’ [10]. Mutations in *XYLT1* were reported in patients with short stature, intellectual disability, flat face and subsequently, *XYLT1* mutations were identified in patients with Desbuquois dysplasia type 2 (OMIM #615777) [11–16]. Deficiency of *XYLT2* causes a spondylo-ocular syndrome (OMIM #605822) with eye and heart defects, hearing loss, fractures and learning problems [17–22]. *B4GALT7* mutations are associated with a rare subtype of the Ehlers-Danlos syndrome (EDS), spondylodysplastic EDS (*B4GALT7*-spEDS; OMIM #130070), characterized by short stature, joint hypermobility, hyperelastic skin, osteopenia and ocular problems [23–28]. A specific homozygous *B4GALT7* mutation, c.808C > T p.(Arg270Cys), is associated with the Larsen of Reunion Island syndrome, characterized by severe joint hypermobility with dislocations [29]. Biallelic mutations in *B3GALT*6 also cause a spectrum of overlapping disorders. Malfait et al. [30] described a subtype of EDS (spondylodysplastic EDS, *B3GALT6*-spEDS) with skin fragility, delayed wound healing, joint laxity/contractures, intellectual disability and spondyloepimetaphyseal dysplasia, whereas Nakajima et al. [10] described a group of individuals suffering from spondyloepimetaphyseal dysplasia with joint laxity type 1 (SEMD-JL1; OMIM # 271640). Van Damme et al. [31] expanded the spectrum of *B3GALT6*-spEDS with cardiac defects, cervical spine instability, respiratory insufficiency and cerebrovascular accidents. Baasanjav et al. [32] reported the first biallelic *B3GAT3* mutations in a consanguineous family and hitherto, *B3GAT3* mutations have been reported in 24 patients from 11 families, all displaying multisystemic conditions characterized by craniofacial, cardiovascular and skeletal abnormalities [32–39].

Here, we report the clinical and molecular findings in two new independent patients with biallelic *B3GAT3* mutations and we provide a literature overview of the genetic and phenotypic findings in all hitherto reported *B3GAT3* patients. We also performed structural modelling of the reported missense mutations in *B3GAT3* to investigate possible genotype-phenotype correlations. Our findings contribute to a better knowledge on the genotypic and phenotypic spectrum of *B3GAT3*-related disease and its delineation from the other linkeropathies.

## Results

### Review of literature

Mutations in *B3GAT3* are extremely rare, with only 23 reported patients from 10 families harboring biallelic *B3GAT3* mutations and one patient with a heterozygous *B3GAT3* mutation [32–39]. Consistent clinical findings in patients with biallelic *B3GAT3* mutations are: skeletal dysplasia (present in all) with shortening and bowing of long bones, (kypho)scoliosis, foot deformity and radioulnar synostosis; disproportionate short stature (16/21, mentioned in 21 of 23 the cases); spatulate distal phalanges (14/15), and facial dysmorphism, present in all patients, albeit with some variability. Characteristic craniofacial features include abnormalities in cephalic index (brachycephaly and dolichocephaly), frontal bossing, hypertelorism, prominent eyes, downslanting palpebral fissures, midface hypoplasia, depressed nasal bridge, microstomia and short neck. Two individuals presented with blue sclerae. Common findings, present in many but not all patients (> 60%), include dislocations of large joints (17/23), contractures of mostly the elbow joint (10/15) and structural cardiac defects, including septal defects and valve abnormalities (12/18). A number of features is more variably present (< 60%). Multiple fractures were reported in 7 patients and joint hypermobility was noted in 8 individuals. Skin involvement is uncommon, with hyperextensible skin, cutis laxa and excessive wrinkling on the palms of the hands, each reported only once [33, 36, 38]. Ocular problems are rare (only present in 3 patients) and diverse, including glaucoma, hyperopia, esotropia, amblyopia and astigmatism [36–38]. Intellectual disability was noted in 2 patients, one of which had multiple brain infarctions and subdural hematomas [37]. Since at least 9 of the families are consanguineous, some rare features are possibly not linked to the *B3GAT3* mutations, but to another homozygous variant. Bloor et al. [34] reported the first patient harbouring a heterozygous splice-site mutation in *B3GAT3*. This patient clearly shared some clinical features associated with biallelic *B3GAT3* mutations, but she also displayed some unique features, including a posterior cloaca, growth hormone deficiency with good response to therapy and sensorineural hearing loss. It is currently not clear whether all these symptoms are related to the *B3GAT3* mutation. We summarized the clinical features in Table 1.

**Table 1:** Summary of clinical features in all reported patients with biallelic B3GAT3 mutations

	Previously reported patients						New patients		Total^c^
	c.830G > A [32, 38]	c.419C > T [35]	c.1A > G + c.671 T > A [36]	c.245C > T [33]	c.667G > A [37, 39]	Het. c.888 + 262 T > G [34]	1. c.667G > A	2. c.416C > T	
N° of patients	6	8	1	1	7	1	1	1	26
Short stature	6/6 (>p3)	8/8 (>P3)	0/1	1/1	1/4 (NR in 3)	1/1	Yes	Yes	19/23 (83%)
Skeletal dysplasia^a^	6/6	8/8	1/1	1/1	7/7	1/1	Yes	Yes	26/26 (100%)
Joint hypermobility	6/6	0/8	1/1	NR	0/1 (NR in 6)	1/1	Yes	Yes	10/19 (53%)
Joint dislocations	6/6 (elbow, shoulders, radioulnar, hip)	8/8 (elbow, shoulder)	1/1 (left hip)	NR	3/7	0/1	No	Yes	18/25 (72%)
Fractures	NR	NR	1/1 (multiple fractures of femur and tibia)	1/1	5/6	0/1	Yes	No	8/12 (67%)
Joint contractures	5/6 (elbow)	4/8 (elbow)	NR	NR	7/7	NR	Yes	No	11/16 (69%)
Facial dysmorphology^b^	6/6	8/8	1/1	NR	6/7	1/1	Yes	Yes	25/25 (100%)
Cardiovascular involvement	6/6 (bicuspid aortic valve, aortic root dilatation, mitral valve prolapse, ASD, VSD)	0/3 (Not investigated in 5)	1/1 (PFO, bicuspid aortic valve, diltation of aortic root and ascendig aorta)	NR	4/7 (ASD, VSD, patent ductus arteriosus)	1/1 (VSD, pulmonary stenosis)	No	NI	12/20 (60%)
Intellectual disability	1/6	0/5	1/1	NR	1 (NR in 6)	NR	–	No	2/14 (14%)
Ocular involvement	1/1 (hyperopia, esotropia, amblyopia)	NR	1/1 (hyperopia, astigmatism, amblyopia and left ptosis)	NR	1 with bilateral glaucoma (NR in 6)	NR	Yes (Corneal clouding)	No	4
Blue sclerae	NR	NR	1/1	NR	1 (NR in 6)	NR	Yes	Yes	4
Spatulate phalanges	6/6	8/8	NR	NR	1 (NR in 6)	NR	Yes	Yes	16/17 (94%)
Hyperextensible skin	0/1 (NR in 5)	NR	1/1	NR	0/7	NR	No	No	1
Cutis laxa	NR	NR	NR	1/1	NR	NR	Yes	No	2
Hearing loss	NR	NR	NR	NR	1 with bilateral conductive (NR in 6)	1/1 (sensorineural)	NI	No	2
Aditional features	Excessive wrinkling of the skin in 1		Restrictive lung disease due to scoliosis + macrocepahly + hypoglycemia + hypothyroidism	Multiple bony chondroma	6 patients died before the age of 1 year	Posterior cloaca + ketotic hypoglycemia + GH deficiency	Died before the age of 1 year		

*NR* Not Reported, *NI* Not Investigated

^a^ Skeletal dysplasia including shortening and bowing of long bones, severe (kypho)scoliosis, foot deformity and radioulnar synostosis

^b^ Facial dysmorhpology including abnormalities in cephalic index (brachycephaly and dolichocephaly), frontal bossing, hypertolerism, prominent eyes, downslanting palpebral fissures, midfacial hypoplasia, depressed nasal bridges, microstomia and short neck

^c^ Total based on the reported frequency

### Case reports

#### Patient 1

**Fig. 1:**
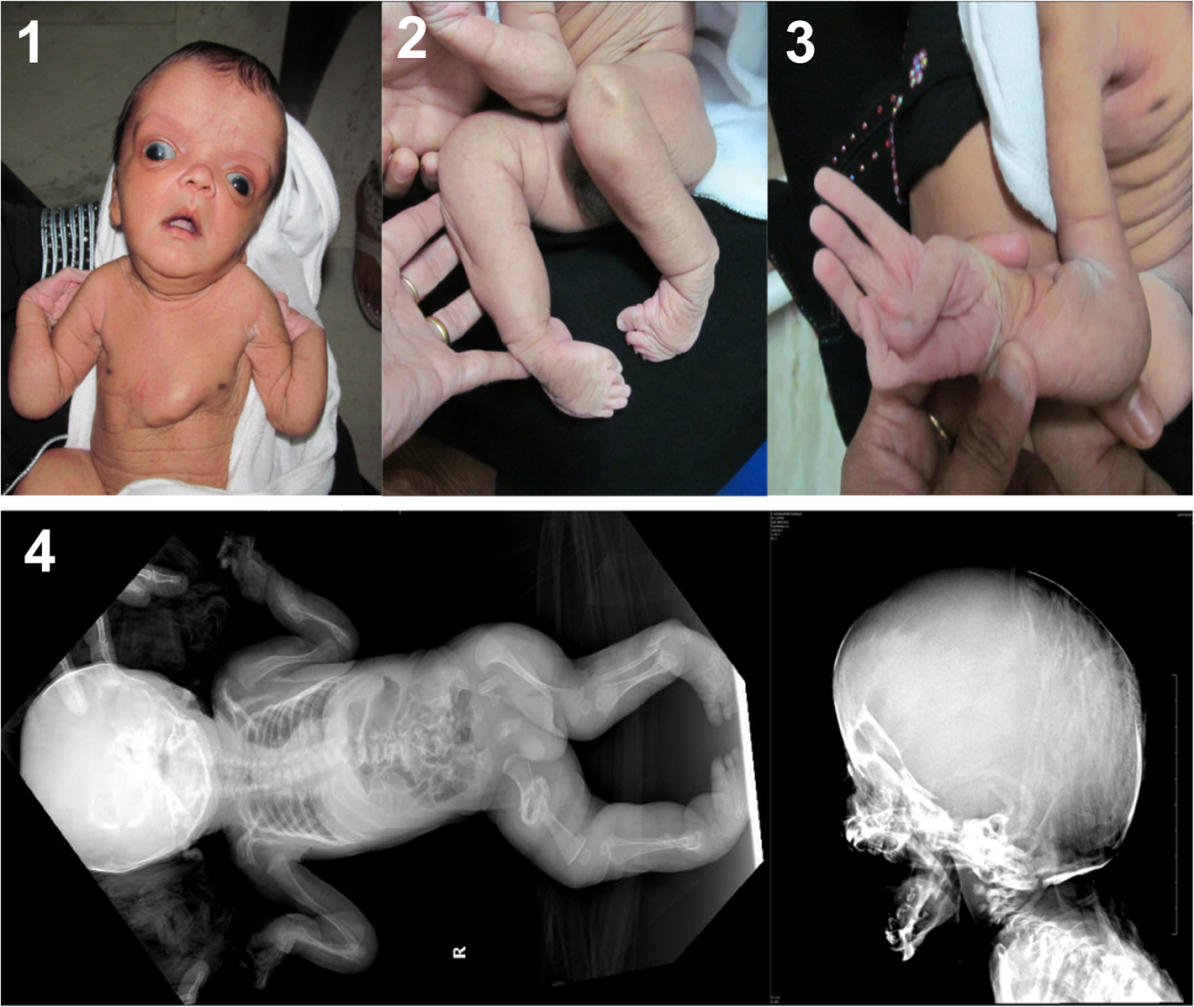
Patient 1 with severe dysmorphic features. There is bulging of the thoracic cage, hypertolerism, downslanting palpebral fissures, lagophtalmos of the lower eye lids, blue sclerae, a pug nose, low-set and dysplastic ears, corneal clouding [1], generalized cutis laxa [1–3], long fingers with campylodactyly and adducted thumbs, broad tips of fingers and toes and bilateral club feet [2, 3]. Perinatal radiography shows osteopenia, multiple fractures, large joint contractures and Wormian bones in the occipital region [4]

Patient 1 is an Indian boy and third child from a third-degree consanguineous marriage. His parents and two older siblings were reportedly healthy. His birth length was 46 cm (<P3), birth weight 2600 kg (P5-P10), and the occipitofrontal circumference 37 cm (P75). He had a large anterior fontanel, which communicated with the posterior fontanel. At the moment of examination 19 days after birth (Fig. 1), he had contractures of both large and small joints (including bilateral talipes equinovarus, flexion contractures of elbows, hips and knees), broad tips of fingers and toes, adducted thumbs and long fingers with camptodactyly of digits 3–5 of the left and digit 5 of the right hand. In addition, he had a short neck and a severely asymmetrical thoracic cage with bulging of the left thoracic side. His skin was cutis laxa-like with excess skin folds, especially over the dorsum of hands and feet and the frontal region. He also displayed severe facial dysmorphic features such as dolichocephaly, hypertelorism, large eyes, downslanting palpebral fissures, lagophtalmos of the lower eye lids, blue sclerae, a pug nose, low-set and dysplastic ears and microstomia with a high-arched palate. Ophthalmological examination revealed bilateral corneal clouding with sclerocornea in the upper parts. His radiographs showed gracile long bones with a thin cortex and multiple fractures and some wormian bones in the occipital region. There was no cardiovascular involvement. He died at the age of 2.5 months because of an unknown reason. No autopsy was performed.

#### Patient 2

**Fig. 2:**
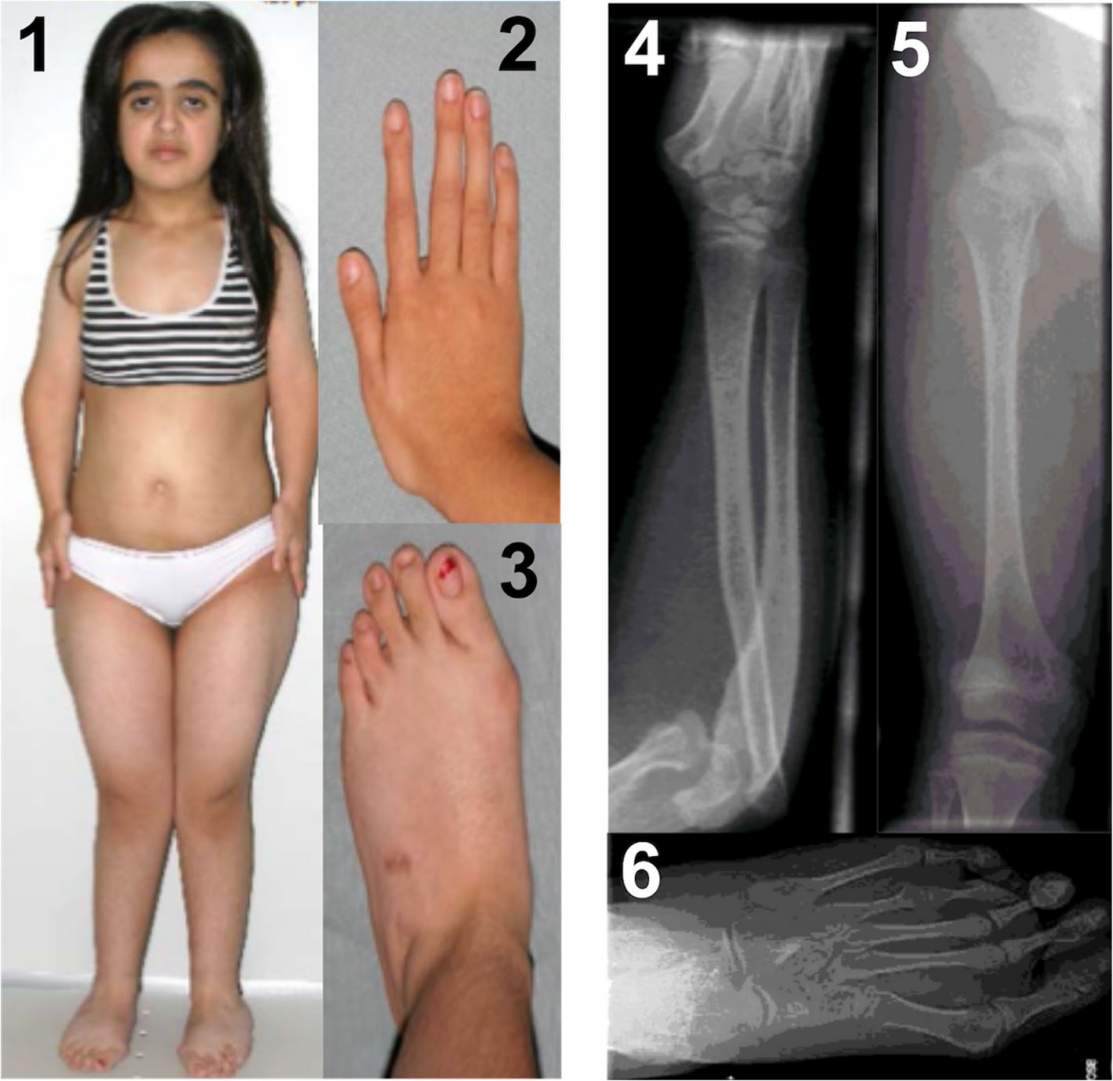
Patient 2 at age 13 yrs. with a disproprionate short stature, genu valgum, a round flat face with midfacial hypoplasia, blue sclerae, downslanting palpebral fissures and prominent eyes [1]. She has long fingers with spatulate distal phalanges and pes planus with hallux valgus [2, 3]. Radiography shows radial head dislocation, short femoral neck, subluxation of the knee joint and irregular tarsal bones [4–6]

Patient 2 is a Turkish girl, the first child of consanguineous parents. She was born at 37 weeks of gestation with a birth weight of 2140 g and length of 43 cm (both <P3). Final height was 129,5 cm (− 5,3 SDS) with a weight of 37 kg. When examined at the age of 13 years (Fig. 2), she presented with a disproportionate short stature, short arms and a very short neck. Her face was round with midfacial hypoplasia, prominent eyes, downslanting palpebral fissures, blue sclerae, a long philtrum, a bifid uvula and a high-arched palate. She displayed generalized joint hypermobility, bilateral radial head dislocation, genua valga, pes planus and hallux valgus. Her fingers had a tapered aspect with spatulate distal phalanges. Motor development was mildly delayed but intelligence was normal. Bone mineral density at the age of 18 years was low with a Z-score of − 1,8 at L1-L4, without fractures. Radiographic imaging showed radial head dislocation, subluxation of the knees, a short femoral neck and irregular tarsal bones. Cardiac investigations were not performed.

### Molecular and structural analyses

**Fig. 3:**
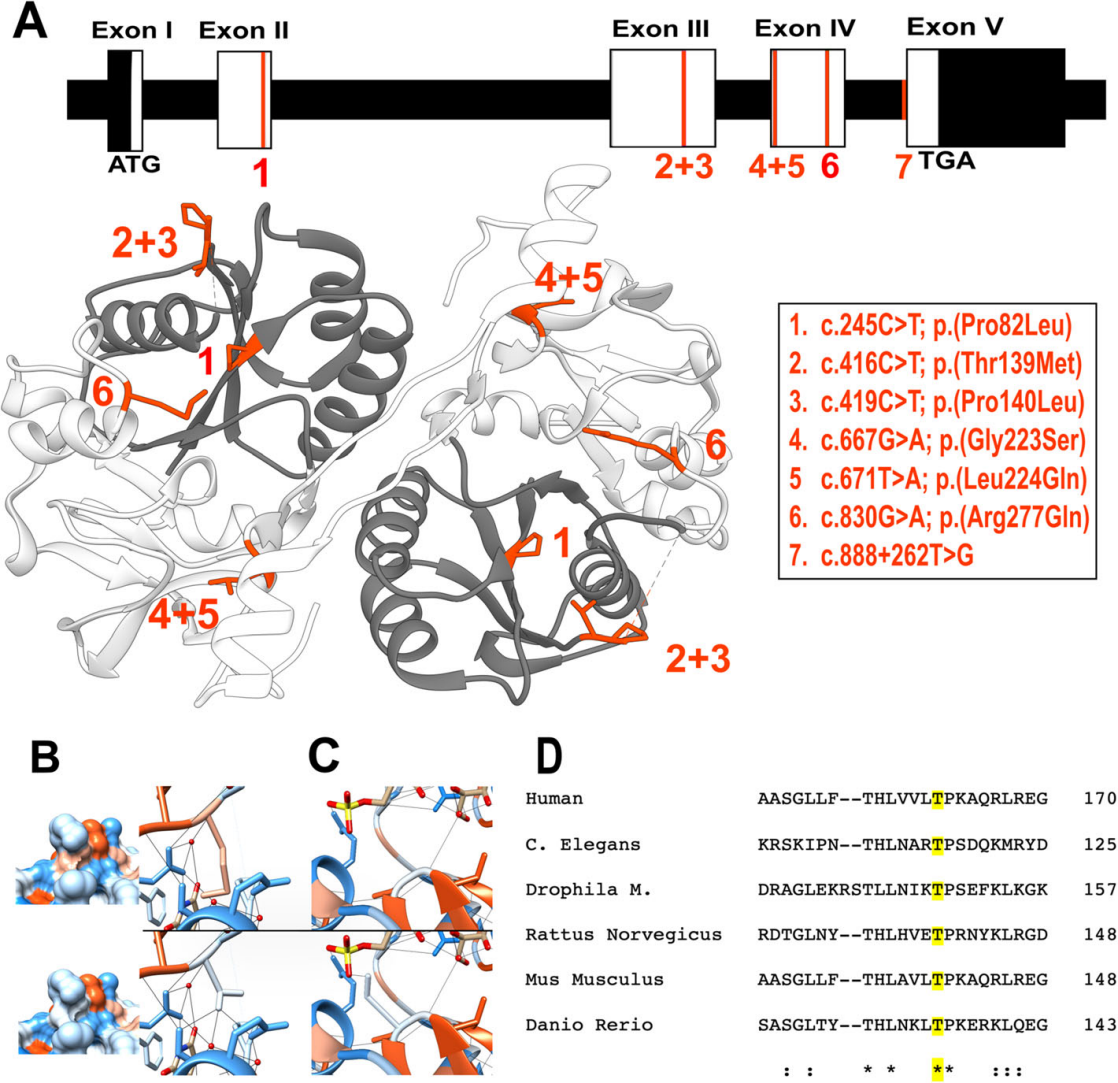
**a** Overview of all the known pathogenic mutations on a schematic representation of the cDNA and gDNA transcript of *B3GAT3* on top and a representation of all missense mutations on an in silico model of GlcAT-I in which the substrate donor is colored in dark grey and the substrate acceptor subdomain is colored light grey. All mutations are highlighted in red. **b**-**c** In silico modelling. Hydrophilic residues are blue, hydrophobic residues are red. **b** p.Thr139 (top row) and the p.(Thr139Met) variant (below) with hydrophobicity surface rendering showing a change in the missense variant. The right column shows the disruption of 3 H-bonds in the missense variant. **c** The p.Gly223 residue (on top) and the p.(Gly223Ser) variant (below) showing the formation of a new H-bond. **d** Clustal Omega protein sequence aligment showing that the protein sequence of GlcAT-I is (largely) conserved across vertebrates and invertebrates. Asterisks indicate a single, fully conserved residue, colons indicate strong similar properties (> 0.5 on the Gonnet PAM 250 matrix), and periods indicate weak similar properties (< 0.5 in the Gonnet PAM 250 matrix). The sequence alignment shows the high conservation of the Thr residue on position 139 of the sequence (marked in yellow)

Whole exome sequencing in patient 1 identified a homozygous c.667G > A, p.(Gly223Ser) missense variant in *B3GAT3*. This mutation has been reported previously in 7 patients [37] and is absent in the GnomAD database. This variant is predicted to be ‘probably damaging’ (Polyphen-2) and ‘deleterious’ (SIFT), but is regarded as a polymorphism by MutationTaster. Whole exome sequencing in patient 2 revealed a novel homozygous missense variant c.416C > T, p.(Thr139Met) in *B3GAT3.* This variant is found in only 1 of 249,250 alleles (allele frequency 4.012e-6) in the GnomAD database. This residue is highly conserved as shown in Fig. 3 and predicted as pathogenic by Polyphen-2 and MutationTaster.

### Structural modelling of missense variants

To date, 7 *B3GAT3* mutations have been reported, including our newly found variant. There are 6 missense mutations: c.245C > T, p.(Pro82Leu); c.416C > T, p.(Thr139Met); c.419C > T, p.(Pro140Leu); c.667G > A, p.(Gly223Ser); c.671 T > A, p.(Leu224Gln) and c.830G > A, p.(Arg277Gln). All occur in homozygous state, except for the c.671 T > A, p.(Leu224Gln) variant, which was found in compound heterozygosity with a null-variant (c.1A > G), p.(Met1?) [32, 33, 35–39]. Recently, a heterozygeous splice-site variant c.888 + 262 T > G was found in one patient [34]. GlcAT-I, located at the Golgi apparatus membrane, consists of multiple domains: a small cytoplasmic domain (res. 1–7), a transmembrane domain (res. 8–25), a proline-rich stem region (res. 26–74) and a catalytic domain consisting of an UDP-GlcUA (uridine diphosphate – (β-D-)glucuronic acid) donor substrate binding subdomain (res. 75–197) and an acceptor substrate binding subdomain (res. 198–308) [40]. All 5 missense variants are located within the catalytic domain of the protein. We performed structural modelling of these missense variants on the protein structure of GlcAT-I (Fig. 3).

### Mutations in the donor substrate binding subdomain

The in silico model of the p.(Pro82Leu) variant showed a clear overlap in the Van Der Waals forces, indicating possible unfavourable interactions because of a disturbance in the balance of the Van Der Waals forces in this variant. The alteration of threonine to methionine residue in the p.(Thr139Met) variant, identified in our patient 2, was predicted to induce 8 new clashes (close contacts between atoms), disrupt 3 H-bonds and a change of surface hydrophobicity was found. The modelling of the p.(Pro140Leu) variant showed an alteration of the surface hydrophobicity.

### Mutations in the acceptor substrate binding subdomain

The substitution of a glycine by a serine in the p.(Gly223Ser) variant was predicted to cause a new H-bond and 6 clashes. Additionally, a report from Gulberti et al. [41] showed that substitution of p.Gly223 with a bigger alanine residue strongly impaired the enzyme function, suggesting an essential role of this residue for substrate specificity. The p.(Leu224Gln) variant was predicted to cause 4 clashes, and a new overlap between Van Der Waals forces was seen. Due to the localization of the last two variants close to the other protein sequence of the homodimer, they may interfere in the dimerization process. In the p.(Arg277Gln), two new clashes were predicted. In a previously reported mutagenesis experiment, the p.Arg277 residue was confirmed to be essential for the enzymic activity of GlcAT-I [42].

Overall, more alterations were predicted with the variants located in the substrate acceptor subdomain. Also, more literature evidence is available on the importance of the affected residues in this subdomain for enzymatic activity of GlcAT-I (Table 2).

**Table 2:** Results of the in silico modelling of all missense variants in *B3GAT3*

Missense variant	Location	Change in H-bonds	New clashes	Change in surface hydrophobicity	Possible effect on dimerisation	Overlap in Van Der Waals forces	Literature evidence	Total
p.(Pro82Leu)	Substrate donor subdomain	/	/	/	/	+	/	1
p.(Thr139Met)	Substrate donor subdomain	+ (3)	+ (8)	+	/	/	/	3
p.(Pro140Leu)	Substrate donor subdomain	/	/	+	/	/	/	1
p.(Gly223Ser)	Substrate acceptor subdomain	+ (1)	+ (6)	/	+	/	+	4
p.(Leu224Gln)	Substrate acceptor subdomain	/	+ (4)	/	+	+	/	3
p.(Arg277Gln)	Substrate acceptor subdomain	/	+ (2)	/	/	/	+	2

/: no alteration was predicted

## Discussion

Glucuronyltransferase I, encoded by *B3GAT3,* catalyzes the addition of the ultimate sugar residue of the tetrasaccharide linker region between the core protein and the glycosaminoglycan side chains of PG. Homozygous loss-of-function of glucuronyltransferase I causes an heterogenic multisystemic disorder. To date, 23 patients from 10 families with biallelic *B3GAT3* mutations and one patient with a heterozygeous splice-site mutation in *B3GAT3* have been reported. Consistent findings include a skeletal dysplasia with disproprionate short stature, kyphosis, scoliosis and deformity of the long bones, spatulate distal phalanges, and facial dysmorphology. Joint hypermobility, joint dislocations/contractures, bone fragility and congenital heart defects are variably present. Other, more infrequent features are ocular problems, intellectual disablilty and skin abnormalities. The two new unrelated individuals we present here showed a clear phenotypic overlap with the previously reported patients. Patient 1, harbouring the p.(Gly223Ser) variant, presented with a severe phenotype including contractures of both large and small joints, multiple fractures, an asymetric thoracic cage, severe dysmorphic facial features, cutis laxa and corneal clouding, the latter being a unique finding. He died at the age of 2.5 months. Patient 2, in which a novel p.(Thr139Met) variant was found, displayed a milder phenotype with short stature, facial dysmorphic features, joint hypermobility and spatulate distal phalanges.

To date only 7 *B3GAT3* mutations have been identified. Although the total number of patients with *B3GAT3* mutations is still very small, some genotype-phenotype patterns start to emerge. All patients harbouring the c.667G > A, p.(Gly223Ser) substitution, including our patient 1, have a strikingly severe phenotype with a high mortality soon after birth. Also the phenotype of the patients harbouring the c.830G > A, p.(Arg277Gln) variant and the compound heterozygous mutation c.[1A > G];[671 T > A], p. [(Met1?)]; [(Leu224Gln)] seem to be at the severe end of the spectrum. In contrast, our patient 2 with c.416C > T, p.(Thr139Met) displays a milder phenotype, as do the affected members of the family harbouring c.419C > T, p.(Pro140Leu) mutations and the individual with the heterozygeous splice site mutation c.888 + 262 T > G. The clinical data available of the patient with the c.245c > T, p.(Pro82Leu) variant is too limited to draw any conclusions about the severity of the phenotype. Data on functional consequences of these mutations was only reported on 3 of the 7 reported mutations. Compared with wild type, the enzyme activity of the p.(Leu224Gln) and p.(Arg277Gln) variants was reduced to 3–5% and in the p.(Pro140Leu) variant, possibly associated with a milder phenotype, a residual enzyme activity to 10% was detected. In the p.(Leu224Gln) and p.(Arg277Gln) variants, a significant reduction of GlcAT-I protein levels was noted in patient fibroblasts, with normal levels of mRNA. No significant differences were found in protein levels when using a recombinant cell system in which vectors of the p.(Pro140Leu) and the p.(Arg277Gln) variants were transfected. This suggests a decrease in activity of the mutant protein affecting the stability of the protein rather than a reduced production. In all 3 variants the amount of CS and HS chains was reduced, indicating a disruption of the effective GAG synthesis [32, 35, 36]. In order to gain insight in the molecular consequences of the reported missense mutations on protein structure, we performed in silico modelling. Strikingly, all missense variants associated with a more severe phenotype are located in the substrate acceptor subdomain of the catalytic domain of GlcAT-I. Overall, more changes in H-bonds and more unfavourable contacs or overlap in Van Der Waals forces were found in the variants located in the substrate acceptor subdomain.

Biallelic mutations have been identified in all five genes coding for the enzymes involved in the synthesis of the tetrasaccharide linker region (Table 3). Each of these linkeropathies is characterized by specific phenotypical patterns, although there is an important overlap. All are characterized by a disproportionate short stature with short limbs, (kypho)scoliosis and an asymmetric/small thorax. Another persistent finding is the presence of facial dysmorphology with a wide spectrum of findings including wide forehead, downslanting palpebral fissures, large eyes, blue sclerae, depressed nasal bridge and midfacial hypoplasia. Excessive joint laxity is present in all disorders, except in *XYLT2* mutations. Joint contractures are only reported in *B3GALT6* and *B3GAT3* mutations, whereas multiple fractures due to bone fragility are commonly found in patients harbouring *B3GALT6* and *XYLT2* mutations and to a lesser extent in mutations in *B3GAT3, B3GALT7* and *B4GALT7.* Cardiac defects, including septal defects and valve defects, are associated with mutations in *XYLT2, B3GAT3* and, to a lesser extend, *B3GALT6*. Ocular involvement is present in most reported patients with *XYLT2*(cataract and retinal detachment) and *B4GALT7* mutations and is a rare and variable finding in *B3GAT3* mutations. Hearing loss is associated with mutations in *XYLT2* while this is uncommon in the other linkeropathies. Hyperextensible skin is associated with mutations in *B4GALT7* and *B3GALT6* and, as mentioned above, some patients with *B3GAT3* mutations have skin involvement. Intellectual disability is reported in all linkeropathies with a variable frequency. Mild to moderate intellectual disability is present in most individuals with mutations in *B4GALT7* and all patients with mutations in *XYLT1* suffer from a degree of intellectual disability. A recent report by La Croix et al. described the presence of biallelic pathogenic variants in *XYLT1* (including a trinucleotide repeat expansion associated with hypermetylation) in patients diagnosed with Baratela-Scott syndrome (BSS) which is characterized by short stature, facial dysmorphology and intellectual disability [43]. They stated that further detailed phenotyping is necessary to determine whether BSS and Desbuquois dysplasia type II can be distinguished by the presence of intellectual disability or not. Based on our literature overview, an important overlap in clinical features exists with presence of intellectual disability in both.

**Table 3:** comparison between the linkeropathies

	XYLT1 [11–16]	XYLT2 [17–22]	B4GALT7^a^ [23–28]	B3GALT6 [10, 30, 31]	B3GAT3 [32–39]
Short stature	100% (15/15)	53% (9/17)	100% (8/8)	100% (27/27)	83% (19/23)
Skeletal dysplasia^b^	100% (15/15)	94% (16/17)	100% (8/8)	100% (27/27)	100% (26/26)
Joint hypermobility	40% (6/15)	NR	100% (8/8)	88% (22/25)	53% (10/19)
Bone fragility	7% (1/15)	94% (16/17)	62% (5/8)	48% (13/27)	67% (8/12)
Joint contractures	NR	NR	37% (3/8)	59% (16/27)	69% (11/16)
Facial dysmorphology^c^	100% (15/15)	65% (11/17)	87% (7/8)	100% (25/25)	100% (25/25)
Hyperextensible skin/ cutis laxa	NR	NR	87% (7/8)	68% (17/25)	12% (reported in 3)
Cardiovascular involvement	7% (1/15)	35% (6/17)	NR	16% (4/25)	60% (12/20)
Intellectual disability	100% (Present in all older patients)	35% (6/17)	75% (6/8)	20% (5/25)	14% (2/14)
Ocular involvement	NR	88% (15/17)	62% (5/8)	NR	15% (reported in 4)
Hearing loss	7% (1/15)	53% (9/17)	25% (2/8)	NR	8% (reported in 2)

^a^ With exclusion of the Larsen of Reunion Island syndrome cohort from Crathault et al. [23]

^b^ Skeletal dysplasia including shortening and deformity of long bones, (kypho)scoliosis, small thoracic cage, radioulnar synostosis, deformity of the feet

^c^ Facial dysmorhpology including wide forehead, downslanting palpebral fissures, large eyes, blue sclerae, depressed nasal bridge and midfacial hypoplasia

## Conclusion

In summary, loss-of-function mutations in *B3GAT3* are associated with a complex connective tissue phenotype characterized by disproportionate short stature, skeletal dysplasia, facial dysmorphism, spatulate distal phalanges and -to a lesser extent- joint contractures, joint hypermobility with dislocations, cardiac defects and bone fragility. Based on the limited number of reported patients, some genotype-phenotype correlations start to emerge. However, pheno- and genotyping of additional patients with mutations in linkeropathy-associated genes, and study of the spatiotemporal effects of the specific mutations on enzyme activities and GAG synthesis is needed in order to better understand the disease mechanisms and phenotypic outcomes of these linkeropathies.

## Methods

### Case reports

Both patients were referred to a clinical geneticist because of the suspicion of a complex heritable connective tissue disorder. DNA samples were obtained from both patients and their parents. Informed consent was obtained from the patients and parents participating in this study. They consented to the publication of clinical photographs.

### Molecular analyses

Whole-exome sequencing was performed for patient 1 and 2 and their parents using HiSeq 3000 (Illumina) and SOLID 5500 (ThermoFisher), respectively. Reads were mapped and variants were called and annotated with the BCbio pipeline. Variants found in patient 1 were analyzed using our in-house developed analysis platform Seqplorer. The identified variants were confirmed with bidirectional sequencing. Nucleotide numbering reflects cDNA numbering, with + 1 corresponding to the A of the ATG translation initiation codon in the reference sequence (NM_0012200). Amino acid residues are numbered from the first methionine residue of the reference sequence (NP_001275650). Pathogenicity of the variants was evaluated using PolyPhen-2 (http://genetics.bwh.harvard.edu/pph2), MutationTaster (http://www.mutationtaster.org) and SIFT (http://sift.bii.a-star.edu.sg) in patient 1 and with PolyPhen-2 and MutationTaster in patient 2. Occurrence was assessed using the GnomAD database (http://gnomad.broadinstitute.org) [44].

### Structural modeling

Protein structure of GlcAT-I was obtained from the RCSB Protein DataBank (https://www.rcsb.org). The protein structure previously reported by Tone et al. (3CU0) was used as reference [9]. This model contains GlcAT-I in a complex with UDP, Mn^2+^, and Galβ1-3Gal(6-O-sulfate)β1-4Xyl(2-O-phosphate)β1-O-Ser. Visualisation of the protein structure was performed using UCSF Chimera (candidate version 1.13.1, build 41,911) [45]. Using the rotamers function, all missense variants were modelled into the protein structure. The ‘Find H-Bond function’ was used to identify potential hydrogen bonds based on the distance between atoms and possible unfavorable interactions were atoms are too close to each other were identified with visualization of the Van Der Waals forces and the ‘Find Clashes/Contacts function’.

## Data Availability

The data that support the findings of this study are available on request from the corresponding author on reasonable request.

## Abbreviations

EDS: Ehlers-Danlos Syndrome
GAG: Glycosaminoglycans
GlcAT-I: Glucuronosyltransferase I
PG: proteoglycans
